# Paediatric COVID-19 Outcomes: Haematology Parameters, Mortality Rates, and Hospitalization Duration

**DOI:** 10.3390/children10101615

**Published:** 2023-09-28

**Authors:** Abdulrahman Alshalani, Badi A. Alotaibi, Jehad A. Aldali, Hamood AlSudais, Abdulaziz M. Almuqrin, Nasser A. Alshehri, Nasser B. Alamar, Mogtba A. Alhejji

**Affiliations:** 1 Chair of Medical and Molecular Genetics Research, Department of Clinical Laboratory Sciences, College of Applied Medical Sciences, King Saud University, Riyadh 12372, Saudi Arabiaaalmuqrin@ksu.edu.sa (A.M.A.); 2 Department of Clinical Laboratory Sciences, College of Applied Medical Sciences, King Saud Bin Abdulaziz University for Health Sciences, Riyadh 11481, Saudi Arabia; 3 King Abdullah International Medical Research Centre, Riyadh 11481, Saudi Arabia; 4 Department of Pathology, College of Medicine, Imam Mohammad Ibn Saud Islamic University (IMSIU), Riyadh 13317, Saudi Arabia

**Keywords:** COVID-19 outcomes, haematology parameters, peadiatric patients, mortality predictors, coagulation profiles

## Abstract

The global COVID-19 pandemic has strained healthcare systems around the globe, necessitating extensive research into the variables that affect patient outcomes. This study examines the relationships between key haematology parameters, duration of hospital stay (LOS), and mortality rates in COVID-19 cases in paediatric patients. Researchers analyse relationships between independent variables (COVID-19 status, age, sex) and dependent variables (mortality, LOS, coagulation parameters, WBC count, RBC parameters) using multivariate regression models. Although the R-square values (0.6–3.7%) indicate limited explanatory power, coefficients with statistical significance establish the impact of independent variables on outcomes. Age emerges as a crucial predictor of mortality; the mortality rate decreases by 1.768% per age group. Both COVID-19 status and age have an inverse relationship with length of stay, emphasising the milder hospitalisation of children. Platelet counts decline with age and male gender, potentially revealing the influence of COVID-19 on haematological markers. There are significant correlations between COVID-19 status, age, gender and coagulation measures. Lower prothrombin time and D-dimer concentrations in elder COVID-19 patients are indicative of distinct coagulation profiles. WBC and RBC parameters exhibit correlations with variables: COVID-19-positive patients have lower WBC counts, whereas male COVID-19-positive patients have higher RBC counts. In addition, correlations exist between independent variables and the red cell distribution width, mean corpuscular volume, and mean corpuscular haemoglobin. However, there is no correlation between mean corpuscular haemoglobin concentration and outcomes, indicating complex interactions between haematological markers and outcomes. In essence, this study underlines the importance of age in COVID-19 mortality, provides novel insights into platelet counts, and emphasises the complexity of the relationships between haematological parameters and disease outcomes.

## 1. Introduction

Coronavirus disease 2019 (COVID-19, COVID), caused by the severe acute respiratory syndrome coronavirus 2 (SARS-CoV-2), has had a devastating effect on the world’s population, resulting in more than 779 million confirmed cases and nearly 7 million deaths worldwide as of August 2023 [1]. The World Health Organization (WHO) declared COVID-19 to be a pandemic in March 2020 after it was first discovered in Wuhan, China, at the end of January 2020. Since then, SARS-2 infections have spread worldwide in tsunami-like waves [2]. Despite the incredible speed with which global mass vaccination initiatives have been conducted, the emergence of new variant strains of SARS-CoV-2 threatens to reverse the progress made, and concerns still need to be addressed about future waves [3]. Several factors contribute to COVID-19 severity and mortality, including older age and comorbidities such as diabetes, kidney disease, hypertension, and cardiovascular disease [4]. Infants and children are expected to exhibit a higher vulnerability to viral infections than adults, and this is primarily attributed to the ongoing development of their immune systems [5]. However, several studies showed fewer severe COVID-19 cases and fatalities among infected children than adults [6,7,8].

Adult and elderly patients were the first documented cases. Children’s data are insufficient because the number of children diagnosed and reported in research is lower than that of adults. Paediatric cases have also been documented as a result of the widespread use of diagnostic tests [9]. As of 20 January 2020, the first paediatric case occurred in China. The 10-year-old’s family had travelled to Wuhan. Although it was infrequently recorded in children during the early phases of the epidemic, as knowledge about COVID-19 grew, it was recognized that children might be infected with SARS-CoV-2 similar to adults [10].

Although children primarily experienced modest manifestations of COVID-19 infection and better prognosis than adults, it is essential to understand that they may still be susceptible to more serious consequences [11,12]. Therefore, identifying laboratory markers that can predict disease severity and mortality in COVID-19 paediatric patients is essential for improving patient management and outcomes. Besides the clinical features, lymphopenia is a haematological finding commonly observed in COVID-19 disease in children and adults [13]. Notably, neutropenia is more commonly reported in paediatric patients with COVID-19 infection than in adults [14,15]. Although neutropenia is a common laboratory finding in some viral infections in children [16], there are limited data on haematological parameters, especially neutropenia, observed in childhood COVID-19 disease patients. Similarly, thrombocytopenia is another hematopoietic systemic abnormality seen in children with COVID-19 [17].

For the clinical management of COVID-19 disease, which can have multiple systemic effects, laboratory parameters are important for diagnosis and treatment monitoring. Although haematological changes in adults have become evident, laboratory findings in children are still unclear because of the small number of studies in children [10]. Since the number of children diagnosed with COVID-19 is gradually increasing, our work is important as the first month of the pandemic is linked to the number of symptomatic children diagnosed. This study examines the haematological parameters of a series of COVID-19 paediatric patients at a Major Saudi Tertiary Center, which could help to develop baseline data for comparison with future SARS-CoV2 infections in children and aid in improving the clinical policy/strategy decisions.

## 2. Materials and Methods

### 2.1. Study Design

The present study was carried out at the King Abdullah International Medical Research Centre (KAIMRC), Ministry of National Guard, Ministry of Health Affairs, Riyadh, Saudi Arabia. The Ministry of National Guard Health Affairs is a government-funded multi-specialty health system that offers primary, secondary, and tertiary treatment [18]. The information was obtained from the BestCare electronic health records system between April 2020 and February 2022. The criteria for selecting the subjects for this study include paediatric patients aged between 1 day and 17 years who had been admitted to the hospital with COVID-like symptoms and were subject to a COVID-19 polymerase chain reaction test immediately. Patients were divided into eight groups according to their PCR COVID-19 test (negative or positive) and age (neonates: birth to 1 month; infants: 1 month to 1 year; children: 1 year through 12 years; and adolescents: 13 years through 17 years).

### 2.2. Patient Data

The research team investigated clinical outcomes including in-hospital mortality and length of hospital stay (LOS). Coagulation parameters such as platelet count, activated partial thromboplastin time (PTT), and D-dimer were also included in the study. In addition, routine haematology laboratory parameters such as white blood cell (WBC) count, red blood cell (RBC) count, and RBC indices which include hematocrit (Hct), mean corpuscular volume (MCV), mean corpuscular haemoglobin (MCH), mean corpuscular haemoglobin concentration (MCHC), and red distribution width (RDW) were captured. All of these data were recorded and included in an Excel sheet. The ethical approval for the study was obtained from the institutional review board at KAIMRC under the approval number (IRB/1266/22).

### 2.3. Statistical Analysis

Statistical analysis was performed using SPSS software (version 26) and graphical presentations were created with GraphPad Prism (version 9.4.1). The normality of the data was assessed using the Shapiro–Wilk test. Unless stated otherwise, data were summarized using medians and interquartile ranges (IQR) for continuous data or percentages and frequencies for categorical data. Since the study has eight groups, data were analysed using a one-way ANOVA test for normally distributed data; otherwise, a Kruskal–Wallis test was used to identify significant differences between study groups. Logistic regression (for mortality outcome) and multiple linear regression (for LOS, WBC, RBC indices, and coagulation outcomes) were conducted in order to assess the relationship between outcomes and independent variables of the COVID-19 test, sex, and age. A *p*-value of less than 0.05 was considered significant.

## 3. Results

### 3.1. Study Population

Table 1 presents the summary statistics for the study groups. A total of 9143 paediatric patients admitted to the hospital with COVID-like symptoms were included in the study. Of those, there were 7748 COVID-19-negative individuals; 343 neonates, 755 infants, 4547 children, and 2103 adolescents. The remaining 1395 patients were COVID-19-positive individuals; 17 neonates, 80 infants, 724 children, and 574 adolescents. Approximately, the study population (49.5%; N, 4526) was females, and (50.5%; N, 4617) was males. Length of hospital stay (LOS) was significantly higher in neonates and infant COVID-19-negative groups with medians of 7 (IQR 4–17) and 2 (1–10), respectively, compared to other study groups (*p*-value < 0.001). The mortality rate was significantly higher in the neonate negative group (4.7%; N, 16) and neonate positive group (5.9%; N, 1) compared to other study groups (*p*-value < 0.001).

### 3.2. Impact of COVID-19 on Haematology and Coagulation Parameters

**Figure 1:**
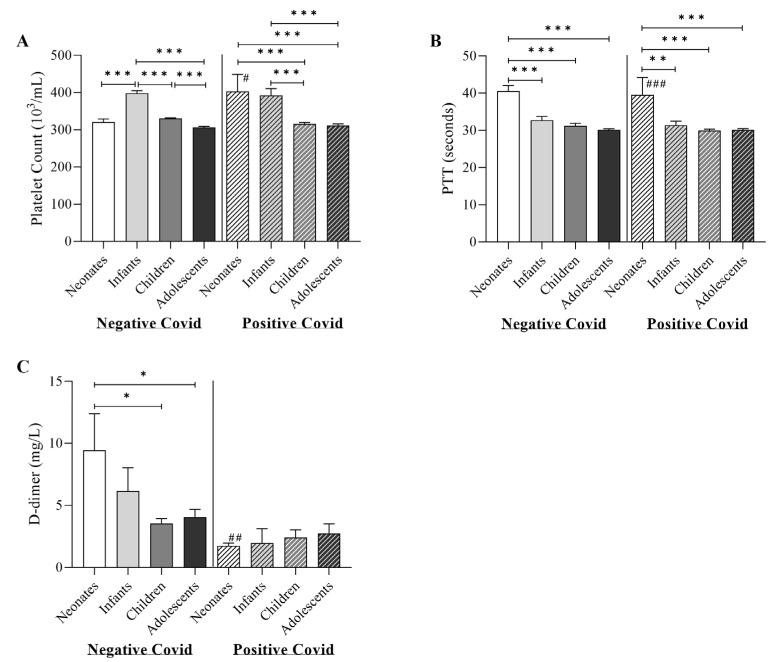
Differences of the study groups in coagulation parameters of (**A**) platelets count; (**B**) PTT (partial thromboplastin time); and (**C**) D-dimer (fibrinogen degradation products). * denotes a significant difference (* *p* < 0.05, ** *p* < 0.01, *** *p* < 0.001). ^#^ denotes a significant difference from the same age group of COVID-19-negative patients (^#^ *p* < 0.05, ^##^ *p* < 0.01, ^###^ *p* < 0.001).

**Figure 2:**
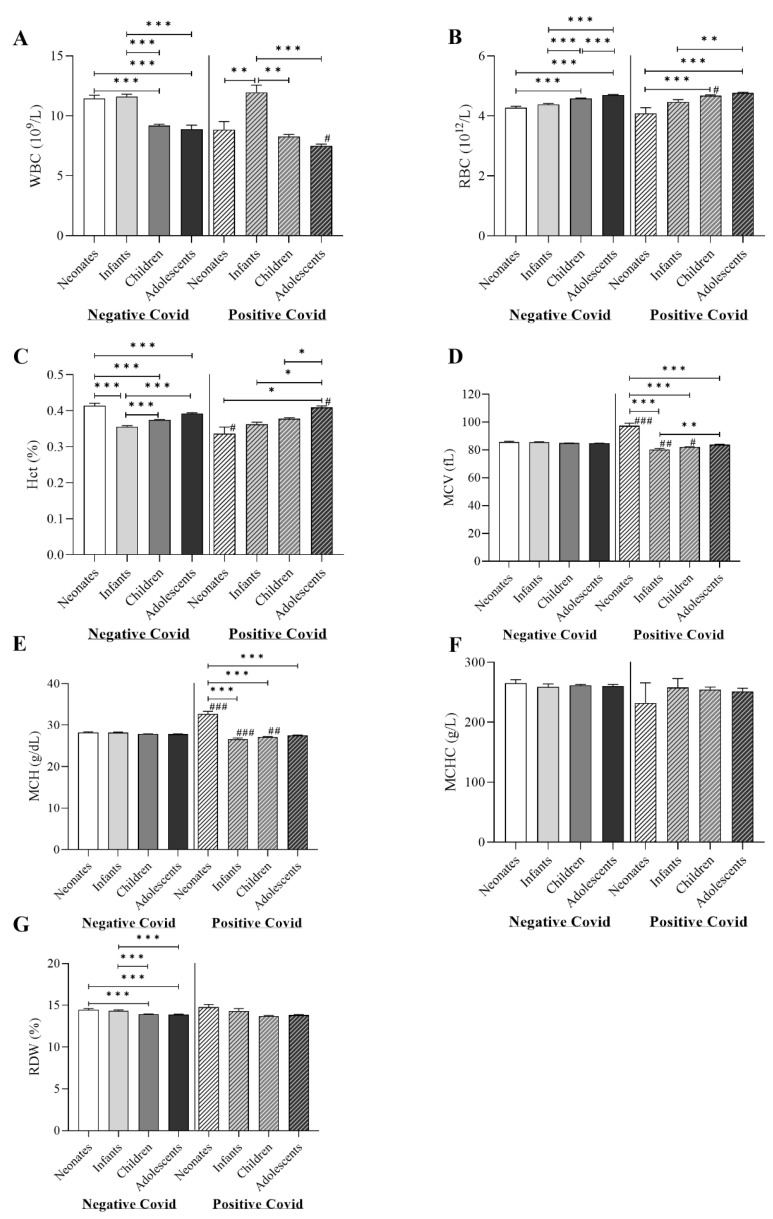
Differences of the study groups in WBC and RBC parameters of (**A**) WBC count; (**B**) RBC (red blood cell) count; (**C**) Hct (hematocrit); (**D**) MCV (mean corpuscular volume); (**E**) MCH (mean corpuscular haemoglobin); (**F**) MCHC (mean corpuscular haemoglobin concentration); and (**G**) RDW (red cell distribution width). * denotes a significant difference (* *p* < 0.05, ** *p* < 0.01, *** *p* < 0.001). ^#^ denotes a significant difference from the same age group of COVID-19-negative patients (^#^ *p* < 0.05, ^##^ *p* < 0.01, ^###^ *p* < 0.001).

The coagulation and haematology parameters were compared between study groups as shown in Figure 1 and Figure 2. Our results revealed a significant increase in platelet count and PTT with a significant decrease in D-dimer level in the COVID-19-positive neonate group (360 × 10^3^/mL, IQR; 249–564, 38.50 s, IQR; 30.25–44, and 1.8 mg/L, IQR; 1.3–2.2, respectively) compared to the COVID-19-negative neonate group (300 × 10^3^/mL, IQR; 216–411, 35.8 s, IQR; 30.6–43.2, and 4.4 mg/L, IQR; 0.9–17.5, respectively) (*p*-value of <0.05, <0.001, and <0.01, respectively) (Figure 1). Within COVID-19-negative groups, the analysis revealed that the infant group was associated with significantly higher platelet count (377 × 10^3^/mL, IQR; 285–476.3) compared to other COVID-19-negative groups (*p*-value < 0.001). In the COVID-19-positive groups, neonates and infants groups showed significantly higher platelet count (360 × 10^3^/mL, IQR; 249.5–564 and 372.5 × 10^3^/mL, IQR; 284–377, respectively) compared to children and adolescents groups (311.5 × 10^3^/mL, IQR; 246.8–377 and 300 × 10^3^/mL, IQR; 244.5–370, respectively) (*p*-value < 0.001). Both COVID-19-negative and COVID-19-positive neonates were associated with significantly higher PTT compared to other groups (*p*-value < 0.001). For the level of D-dimer, neonate COVID-19-negative was associated with a significantly higher level (4.4 mg/L, IQR; 0.9–17.5) compared to children and adolescents COVID-19-positive groups (1.4 mg/L, IQR; 0.6–3.9 and 1.1 mg/L, IQR; 0.4–3.3, respectively) (*p*-value < 0.05).

Figure 2 presents the differences between the study groups in WBC and RBC parameters. Comparing the COVID-19-positive with the COVID-19-negative group, the analysis revealed that adolescent COVID-19-positive patients had significantly lower WBC count (6.8 × 10^9^/L, IQR; 5.2–9) compared to adolescent COVID-19-negative patients (7.4 × 10^9^/L, IQR; 5.7–9.8) (*p*-value of < 0.05). In addition, children COVID-19-positive patients had significantly higher RBC count (4.8 × 10^12^/L, IQR; 4.4–5.1) compared to children COVID-19-negative patients (4.6 × 10^12^/L, IQR; 4.3–4.9) (*p*-value of < 0.05). For hematocrit (Hct), neonate COVID-19-positive patients showed significantly lower Hct (0.33, IQR; 0.30–0.39), and adolescent COVID-19-positive patients showed significantly higher Hct (0.41, IQR; 0.38–0.45) compared to COVID-19-negative patients of the same age group (0.41, IQR; 0.32–0.50 and 0.40, IQR; 0.36–0.44, respectively) (*p*-value of < 0.05). MCV was significantly higher in neonate COVID-19-positive patients (98 fL, IQR; 91.5–104) and significantly lower in COVID-19-positive infants (80.3 fL, IQR; 75.1–84.2) and children (82.7 fL, IQR; 78.6–86.6) compared to COVID-19-negative patients of the same age group (86.9 fL, IQR; 81.1–91.9, 86.3 fL, IQR; 80.2–91.2, and 86 fL, IQR; 80.5–90.7, respectively) (*p*-values of <0.001, <0.01, and <0.05, respectively). Similarly, MCH was significantly higher in neonate COVID-19-positive patients (32.7 g/dL, IQR; 30.8–34.6) and significantly lower in COVID-19-positive infants (26.8 g/dL, IQR; 25.7–27.9) and children (27.5 g/dL, IQR; 26–28.7) compared to COVID-19-negative patients of the same age group (28.5 g/dL, IQR; 26.7–30.1, 28.6 g/dL, IQR; 26.4–30.1, and 28.3 g/dL, IQR; 26.3–29.8, respectively) (*p*-values of <0.001, <0.001, and <0.01, respectively). Within COVID-19-negative and -positive, the analysis revealed that COVID-19-negative neonates and infants had higher WBC counts compared to other negative groups. Both children and adolescents had higher RBC counts within both COVID-19-negative and -positive groups. Hct was significantly higher in COVID-19-negative neonates compared to the other COVID-19-negative groups. Neonates had higher MCV and MCH compared to other COVID-19-positive groups. For RDW, It was higher in COVID-19-negative neonates and infants compared to COVID-19-negative children and adolescents. No significant results were detected in MCHC.

Descriptive data for Figure 1 and Figure 2 are summarized in Supplementary Table S1. In order to assess the effect of patient sex on haematology and coagulation parameters findings, study groups were further split based on patient sex, and independent *t*-tests were carried out Supplementary Figures S1 and S2.

### 3.3. Relationship between Mortality, LOS, and Haematology Parameters in COVID-19 Patients

Table 2 provides the multivariate regression models to illustrate the relationships between the independent variables (COVID-19 status, sex, age) and the dependent variables (mortality, LOS, coagulation parameters, WBC count, and RBC parameters). The models’ R-squared values range from 0.6 to 3.7%, indicating that independent variables were weak in explaining the variance in the dependent variables. In most cases, however, the coefficients for the independent variables are statistically significant, indicating that these variables have some effect on the dependent variables. In detail, there was a significant negative association between age and the mortality rate.

For each increase in the age group, there is a decrease in the mortality rate of 1.768%. Both COVID-19 and age were negatively associated with LOS. For platelet count, there was a significant negative association between both sex and age with platelet count. In detail, for males and each increase in the age group, there is a decrease in the platelet count by 9.881 and 20.170 (10^3^/mL) compared to female and neonate groups, respectively. Moreover, all independent variables included in the model were significantly associated with the differences in PTT. COVID-19-positive patients had a 3.576 (s) increase in PTT compared to COVID-19-negative groups. Male patients were associated with 3.501 (s) decrees in PTT compared to female patients. For each increase in the age group, there is a decrease in PTT of 4.493 (s).

For D-dimer, both the presence of COVID-19 and the age were significantly associated with a decrease in D-dimer level (2.038 and 0.849 mg/L, respectively). Furthermore, the presence of COVID-19 was associated with a 0.999 (10^9^/L) decrease in WBC count compared to negative COVID-19 patients. For each increase in the age group, there is a 1.004 (10^9^/L) decrease in the WBC count. For RBC, all independent variables included in the model were positively associated with RBC count changes. The presence of COVID-19 was associated with a 0.082 (10^12^/L) increase in RBC count compared to negative COVID-19 patients. Male patients had a higher RBC count of 0.159 (10^12^/L) compared to female patients. For each increase in the age group, there is a 0.148 (10^12^/L) increase in the RBC count. 

Similarly, all independent variables included were positively associated with Hct changes in a model explaining 1.0% of variation in Hct. In MCV, the presence of COVID-19 and sex variables were significantly associated with changes in MCV. The presence of COVID-19 was associated with a 2.066 (fL) decrease in MCV compared to negative COVID-19 patients. Male patients had a higher MCV of 0.863 (fL) compared to female patients. All independent variables included in the model were significantly associated with MCH and RDW changes. No significant associations were detected between MCHC and independent variables included in the regression model.

## 4. Discussion

The ongoing global pandemic caused by the novel coronavirus (COVID-19) has led to a significant burden on healthcare systems worldwide. Researchers and medical professionals have been striving to understand the intricate relationship between various clinical and demographic factors and COVID-19 outcomes. One crucial aspect of this understanding involves investigating the link between mortality rates, length of hospital stay (LOS), and key haematology parameters in COVID-19 patients.

This study employs multivariate regression models to investigate the potential associations between independent variables (COVID-19 status, sex, age) and dependent variables (mortality, LOS, coagulation parameters, WBC count, and RBC parameters) with a focus on paediatric patients. The range of R-squared values between 0.6% and 3.7% indicates that the independent variables have a limited capacity to explain the observed variance in the dependent variables. Despite this, the statistically significant coefficients indicate that the independent variables do influence the investigated outcomes. In addition, the study uncovers a noteworthy negative association between age and the mortality rate among COVID-19 patients. Interestingly, for each rise in the age group, there is a corresponding decrease in the mortality rate by 1.768% (Table 2). This highlights the importance of age as a significant predictor of COVID-19-related mortality. Furthermore, both COVID-19 status and age display a negative association with LOS, emphasising the observed lower effect of COVID-19 on paediatric hospitalization. 

This disparity in mortality rates could be attributed to differences in healthcare adequacy, patients’ epidemiological factors, and the frequency of diagnostic screening in asymptomatic or mildly symptomatic patients [19]. However, regardless of geographic region, there is a consistent and evident trend of an age-based exponential increase in fatality rate. The overall case fatality rate (CFR) was 2.37% in 11,344 patients with confirmed cases on 28 May 2020, according to the Korea Centers for Disease Control and Prevention, but it was substantially higher in the elderly (10.9% in patients aged 70–79 years and 26.6% in patients 80 years) [19]. COVID-19 fatality varies from other respiratory viral illnesses in that the severity pattern is generally described as a U-shaped curve, with morbidity and mortality concentrated at extreme age groups (younger children and the elderly).

Several cohort studies have found that the hospitalization incidence for influenza was higher for children aged 0 to 4 years compared to other age groups, implying that children under the age of 5 years may be more prone to influenza and/or more severely impacted, as the hospitalization fatality rate for children aged 0 to 4 years was quite low [20,21]. This age group’s peak on the mortality curve, which was based on 100,000 people, was substantially lower than that of the elderly. However, this kind of mortality peak has not yet been seen in children with COVID-19, which raises the possibility that these children are less prone to the illness or have milder symptoms and a smaller CFR than children infected with influenza.

Interesting new insights into the fluctuating platelet count are uncovered by our investigation. Platelet counts are inversely related to both advancing age and male gender. This discovery may help shed light on the pathogenesis of COVID-19 and its effect on haematological markers. Although variations in platelet count and function are assumed to be multifactorial during the disease, three primary ideas have been proposed. The first is bone marrow suppression caused by infection and a decrease in platelet production; the second is platelet destruction caused by an increased immune response; and the third is platelet consumption caused by the formation of microthrombi in the lungs and other organs [22].

Our study showed significant correlations between COVID-19 status, sex, age and coagulation measures such as prothrombin time and D-dimer levels. An elevated PTT might indicate possible coagulation abnormalities in COVID-19 patients. Patients’ PTT values are lower in males than in females, and they continue to drop with age. Similarly, older patients and those with COVID-19 have lower D-dimer levels, suggesting that the coagulation profile of COVID-19 patients may differ from the norm. Our data, while not statistically significant in groups other than neonates, showed decreased D-dimer levels in PCR-confirmed COVID-19 patients as compared to negative COVID-19 patients. The association between COVID-19 severity and elevated levels of D-dimer in children has been reported [23]. It is important to note that elevated D-dimer levels can be a consequence of lung injury that is seen in several respiratory conditions such as pneumonia [24]. All subjects included in our study have COVID-19-like symptoms, which suggests that the non-COVID-19 subjects might have other respiratory diseases that can elevate D-dimer levels because of acute lung injury [24]. The evaluation of D-dimer levels in various respiratory diseases as compared to COVID-19 is yet to be fully investigated. In our data, 75% of all subjects (COVID-19-positive and negative) have a D-dimer level ≥ 1.8 (mg/L), which is high (Supplementary Table S1). Further investigation is needed for better evaluation of the observed lower D-dimer levels in COVID-19 patients as compared to non-COVID-19. Contrary to conventional sepsis, individuals with COVID-19 frequently have normal PTTs, with only 6% of patients experiencing PTT prolongation [25]. In COVID-19 critically ill and non-critically ill individuals, the average aPTT duration seems to be similar, with no discernible relationship to disease severity or mortality [25].

WBC and RBC parameters also show substantial relationships with the variables under consideration. COVID-19-positive patients display decreased WBC counts, and increasing age is associated with declining WBC counts, suggesting an impact on the immune response (Figure 2). It has been noted that the clinical and laboratory manifestations in the paediatric age differ from those in adults. The laboratory results of children with COVID-19 are consistent with other coronavirus infections, e.g., neutropenia and/or lymphopenia are present together with low or abnormal WBC counts in children with COVID-19 [10]. A study of children with COVID-19 found that the leukocyte count was normal in 70% of the examined cases, while there was an increase in the leukocyte count in 20% of cases and a drop in the rest of cases [26]. In individuals infected with COVID-19, the incidence of leukocytosis (neutrophilia and/or lymphocytosis) is lower, and it is a precursor to bacterial infection or superinfection in particular [10].

On the other hand, RBC parameters demonstrate positive associations with all independent variables, with COVID-19-positive patients and male individuals exhibiting increased RBC counts. There are substantial correlations between numerous independent variables and the red cell distribution width (RDW), mean corpuscular volume (MCV), and mean corpuscular haemoglobin (MCH). Male patients tend to have greater MCV values than female patients, and the presence of COVID-19 is associated with lower MCV levels. Changes in MCH and RDW are correlated with all independent variables, suggesting that COVID-19 may be linked to haematological abnormalities. MCHC and the regression model’s independent variables are not significantly correlated. This suggests that MCHC may not be a good predictor of COVID-19 results due to the complexity of the interaction between haematological markers and outcomes.

It is important to mention that the sample of neonates that were COVID-19-positive was quite small, with only 17 patients. With a small sample size, caution for reporting false negative results for COVID-19-positive neonates must be applied, as the small sample size of this group reduces the statistical power to detect any significant differences from other groups. Therefore, further investigations are needed to confirm the conclusions made during this work. Unlike other reports, individuals with severe COVID-19 had significantly lower hematocrit and haemoglobin values compared to those with mild/moderate disease (both *p* 0.001), whereas RDW values increased with increasing severity (*p* 0.001). Similar effects were obtained at the maximum 30-day severity. Hematocrit and haemoglobin levels decreased considerably with increasing severity (*p* = 0.002 and *p* = 0.017, respectively). RDW rose progressively with severity (*p* = 0.001) [27]. 

Finally, we cannot deny that our study has a few limitations. First, it is retrospective research that, first and foremost, addresses mild and asymptomatic cases. Second, retrospective collection, rather than daily testing, was used to gather the laboratory results for the patients at the time of admission. Patients were admitted at various points in the disease’s progression, and these laboratory results can change over time.

## 5. Conclusions

The results of this study provide important insight into the complex relationship between mortality, length of hospital stay, and a variety of haematological markers in patients with COVID-19. Although the independent variables’ R-squared values suggest only a moderate ability to explain the results, the coefficients that reach statistical significance highlight their influence. These results add to our knowledge of the complex nature of COVID-19 and may help inform clinical care and future investigations.

## Figures and Tables

**Table 1:** Characteristics of patients included in the study.

Variable	Groups								*p*-Value
	Negative COVID-19				Positive COVID-19				
	Neonates	Infants	Children	Adolescents	Neonates	Infants	Children	Adolescents	
N	343	755	4547	2103	17	80	724	574	--
Sex, N (%)									--
Female	169 (49.3)	322 (42.6)	2325 (51.1)	1011 (48.1)	10 (58.8)	38 (47.5)	330 (45.6)	321 (55.9)	
Male	174 (50.7)	433 (57.4)	2222 (48.9)	1092 (51.9)	7 (41.2)	42 (52.5)	394 (54.4)	253 (44.1)	
LOS, Median (IQR)	7 (4–17)	2 (1–10)	2 (1–5)	3 (1–5)	1 (1–2.25)	1 (1–2)	1 (1–3)	2 (1–3)	<0.001
Mortality, N (%)	16 (4.7)	8 (1.1)	16 (0.4)	8 (0.4)	1 (5.9)	0 (0)	1 (0.1)	1 (0.2)	<0.001

LOS: length of hospital stay, IQR: interquartile range.

**Table 2:** The multivariate regression models for mortality, LOS, and haematology parameters.

Dependent Variables	R Square	Coefficients (B)		
		COVID-19 Negative (Reference)	Sex Female (Reference)	Age Neonates (Reference)
**Mortality**	0.006	−0.757	−0.251	−2.289 ***
**LOS**	0.033	−1.244 ***	−0.169	−1.768 ***
**Coagulation parameters**				
Platelets count	0.013	−4.705	−9.881 ***	−20.170 ***
PTT	0.011	3.576 *	−3.501 **	−4.493 ***
D-dimer	0.019	−2.038 *	0.550	−0.849 *
**WBC count**	0.008	−0.999 ***	0.047	−1.004 ***
**RBC parameters**				
RBC Count	0.037	0.082 ***	0.159 ***	0.148 ***
Hct	0.010	0.007 **	0.009 ***	0.005 ***
MCV	0.011	−2.066 ***	0.863 ***	−0.221
MCH	0.015	−0.523 ***	0.577 ***	−0.126 **
MCHC	0.001	−5.662	2.118	−0.798
RDW	0.018	−0.155 *	−0.587 ***	−0.199 ***

Logistic regression for mortality and linear regression for coagulation parameters. LOS: length of hospital stay; PTT: partial thromboplastin time; D-dimer; fibrinogen degradation products; RBC: red blood cell; Hct: hematocrit; MCV: mean corpuscular haemoglobin; MCH: mean corpuscular haemoglobin; MCHC: mean corpuscular haemoglobin concentration; RDW: red cell distribution width. ^*^ denotes a significant association (* *p* < 0.05, ** *p* < 0.01, *** *p* < 0.001).

## Data Availability

Data can be provided upon request to the corresponding author.

## Supplementary Materials

The following supporting information can be downloaded at: https://www.mdpi.com/article/10.3390/children10101615/s1, Figure S1: Differences in coagulation parameters study groups based on patient sex; Figure S2: Differences in WBC and RBC based on patient sex; Table S1: Descriptive analyses for hematology and coagulation parameters.
