# Supplementary material for: Paediatric COVID-19 Outcomes: Haematology Parameters, Mortality Rates, and Hospitalization Duration
Source: Children (Basel). 2023 Sep 28;10(10):1615. doi: 10.3390/children10101615 (PMC10605890; doi:10.3390/children10101615)
Supplement: Supplementary file 1 [file children-10-01615-s001.zip › children-2595901-supplementary.pdf]

Table S1. Descriptive analyses for hematology and coagulation parameters.

Parameter	Groups							
	Negative Covid				Positive Covid			
	Neonates	Infants	Children	Adolescents	Neonates	Infants	Children	Adolescents
**Platelet count (10^3/mL)**								
Number of values	343	754	4547	2102	17	79	722	574
25% Percentile	216.0	284.5	255.0	242.0	249.5	284.0	246.8	244.5
Median	300.0	377.0	318.0	296.0	360.0	372.0	311.5	300.0
75% Percentile	411.0	476.3	392.0	356.0	564.0	487.0	377.0	370.0
Mean	320.1	398.0	330.1	306.4	402.5	392.3	315.1	311.2
Std. Deviation	167.6	184.9	131.4	115.2	189.9	161.9	116.3	109.0
Std. Error of Mean	9.048	6.734	1.949	2.512	46.07	18.21	4.330	4.548
**PTT (seconds)**								
Number of values	161	308	2079	983	10	27	248	182
25% Percentile	30.55	26.63	26.20	26.70	30.25	27.30	26.20	27.60
Median	35.80	29.40	28.50	29.00	38.50	30.10	28.90	29.40
75% Percentile	43.15	33.85	31.00	31.50	44.00	35.00	32.40	32.00
Mean	40.51	32.68	31.19	30.11	39.50	31.35	29.95	30.15
Std. Deviation	19.25	18.43	32.75	10.07	14.76	5.842	5.741	4.384
Std. Error of Mean	1.517	1.050	0.7182	0.3211	4.669	1.124	0.3645	0.3250
**D-dimer (mg/L)**								
Number of values	16	32	231	134	4	8	39	25
25% Percentile	0.9000	0.8800	0.5700	0.3850	1.275	0.5000	0.5700	0.4500
Median	4.400	2.160	1.410	1.120	1.755	0.7050	0.9000	0.9700
75% Percentile	17.47	3.783	3.980	3.268	2.153	1.820	1.690	3.570
Mean	9.448	6.160	3.548	4.041	1.728	1.954	2.404	2.738
Std. Deviation	11.75	10.50	5.890	7.345	0.4594	3.285	3.945	3.830
Std. Error of Mean	2.938	1.855	0.3876	0.6345	0.2297	1.162	0.6316	0.7659
**WBC (10^9/L)**								
Number of values	343	685	4547	2103	17	80	724	574

25% Percentile	8.380	8.000	6.100	5.700	6.645	8.443	5.400	5.208
Median	10.40	10.70	8.100	7.400	8.900	11.60	7.300	6.845
75% Percentile	13.40	14.05	10.80	9.790	11.25	14.80	9.913	9.000
Mean	11.46	11.60	9.181	8.885	8.849	11.95	8.287	7.507
Std. Deviation	5.405	5.414	8.377	15.12	2.988	5.439	4.531	3.367
Std. Error of Mean	0.2586	0.2069	0.1173	0.3297	0.6681	0.6081	0.1536	0.1405
**RBC (10^{12}/L)**								
Number of values	343	685	4547	2103	17	80	724	574
25% Percentile	3.448	3.990	4.300	4.330	3.310	4.085	4.360	4.400
Median	4.265	4.440	4.630	4.750	4.025	4.515	4.750	4.760
75% Percentile	4.933	4.820	4.950	5.150	4.865	4.943	5.100	5.200
Mean	4.272	4.384	4.582	4.697	4.079	4.466	4.676	4.763
Std. Deviation	1.030	0.7230	0.6514	0.7177	0.8766	0.6756	0.7424	0.7052
Std. Error of Mean	0.04919	0.02762	0.009120	0.01565	0.1960	0.07553	0.02517	0.02943
**Hct (%)**								
Number of values	270	427	2739	1101	7	44	450	280
25% Percentile	0.3210	0.3220	0.3500	0.3555	0.3010	0.3433	0.3548	0.3763
Median	0.4060	0.3600	0.3800	0.3970	0.3270	0.3610	0.3840	0.4140
75% Percentile	0.4970	0.3890	0.4060	0.4370	0.3870	0.3928	0.4090	0.4508
Mean	0.4140	0.3559	0.3747	0.3923	0.3366	0.3626	0.3779	0.4096
Std. Deviation	0.1122	0.05323	0.05459	0.06371	0.04851	0.03763	0.05525	0.06377
Std. Error of Mean	0.006826	0.002576	0.001043	0.001920	0.01834	0.005672	0.002604	0.003811
**MCV (fL)**								
Number of values	343	685	4547	2103	17	80	724	574
25% Percentile	81.08	80.15	80.50	80.90	91.45	75.13	78.60	80.28
Median	86.90	86.30	86.00	86.10	98.00	80.25	82.70	84.95
75% Percentile	91.90	91.20	90.70	90.40	104.0	84.20	86.60	89.20
Mean	85.76	85.51	84.92	84.78	97.34	80.13	82.07	83.87
Std. Deviation	10.23	8.741	8.790	8.721	8.548	8.022	7.204	8.025
Std. Error of Mean	0.4886	0.3340	0.1230	0.1902	1.911	0.8969	0.2442	0.3350

**MCH (g/dL)**								
Number of values	438	685	5104	2103	20	80	870	574
25% Percentile	26.68	26.40	26.30	26.40	30.80	25.70	26.00	26.10
Median	28.50	28.60	28.30	28.30	32.70	26.80	27.50	27.90
75% Percentile	30.10	30.10	29.80	29.80	34.58	27.98	28.70	29.33
Mean	28.22	28.19	27.85	27.82	32.68	26.63	27.16	27.48
Std. Deviation	3.622	3.230	2.986	3.081	2.903	2.431	2.537	2.814
Std. Error of Mean	0.1731	0.1234	0.04180	0.06719	0.6491	0.2718	0.08602	0.1174
**MCHC (g/L)**								
Number of values	438	685	5104	2103	20	80	870	574
25% Percentile	309.0	307.5	308.0	306.0	33.30	103.7	36.08	35.80
Median	325.0	326.0	324.0	323.0	329.5	328.0	325.0	323.0
75% Percentile	334.0	335.0	332.0	333.0	344.5	338.0	335.0	331.0
Mean	265.4	259.0	261.5	260.5	232.0	258.2	254.5	251.6
Std. Deviation	122.0	128.1	123.5	124.5	150.1	131.2	129.5	128.7
Std. Error of Mean	5.831	4.896	1.729	2.714	33.57	14.67	4.390	5.370
**RDW (%)**								
Number of values	438	685	5104	2103	20	80	870	574
25% Percentile	12.70	12.60	12.50	12.50	14.03	12.73	12.60	12.40
Median	13.70	13.70	13.30	13.30	14.35	13.80	13.40	13.30
75% Percentile	15.50	15.40	14.70	14.70	15.90	15.23	14.20	14.50
Mean	14.47	14.35	13.93	13.89	14.78	14.32	13.72	13.82
Std. Deviation	2.883	2.677	2.437	2.470	1.464	2.623	1.858	2.190
Std. Error of Mean	0.1377	0.1023	0.03411	0.05387	0.3273	0.2933	0.06298	0.09142

PTT; activated partial thromboplastin time, WBC; white blood cell count, RBC; red blood cell count, Hct; hematocrit, MCV; mean corpuscular volume, MCH; mean corpuscular hemoglobin, MCHC; mean corpuscular hemoglobin concentration, and RDW; red distribution width.

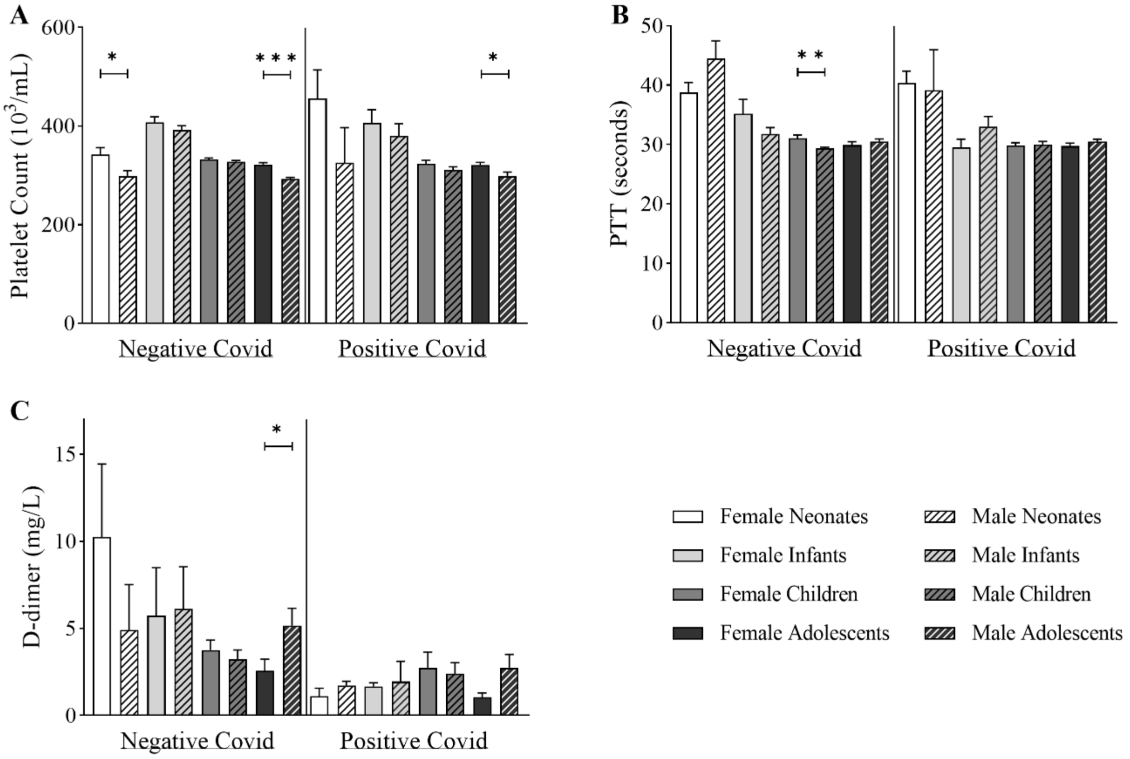

Figure S1. Differences in coagulation parameters study groups based on patient sex. (A) platelets count, (B) PTT (partial thromboplastin time), and (C) D-dimer (fibrinogen degradation products). * denotes a significant difference between females and males (*P < 0.05, **P < 0.01, ***P < 0.001).

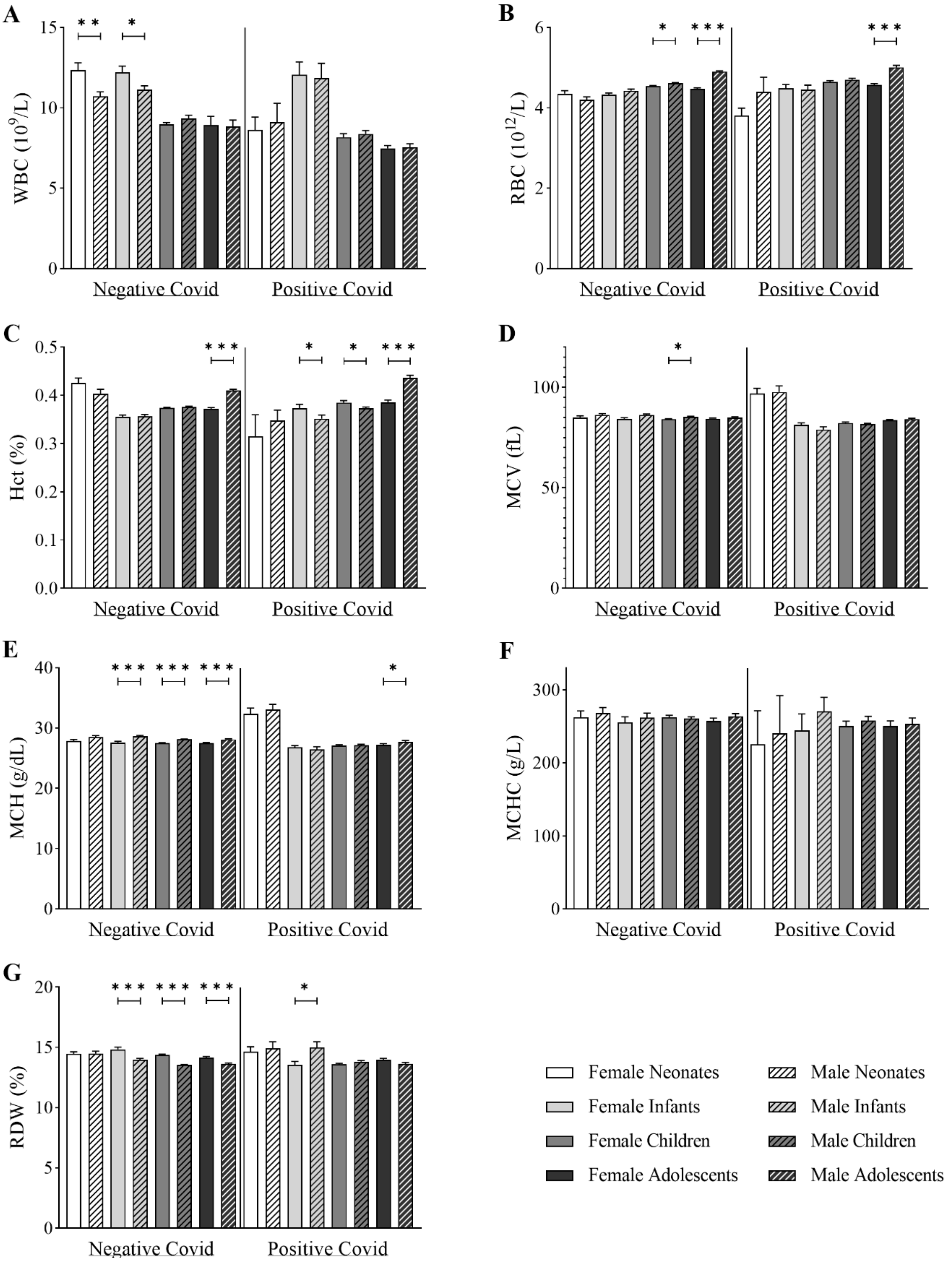

Figure S2. Differences in WBC and RBC based on patient sex. (A) WBC count, (B) RBC (red blood cell) Count, (C) Hct (hematocrit), (D) MCV (mean corpuscular volume), (E) MCH (mean corpuscular hemoglobin), (F) MCHC (mean corpuscular hemoglobin concentration), and (G) RDW (red cell distribution width). * denotes a significant difference between females and males (*$P < 0.05$, **$P < 0.01$, ***$P < 0.001$).
